# Semi-Automated Cell Panning for Efficient Isolation of FGFR3-Targeting Antibody

**DOI:** 10.3390/ijms22126240

**Published:** 2021-06-09

**Authors:** Byeongkwi Min, Minyoung Yoo, Hyeree Kim, Minjung Cho, Do-Hyun Nam, Yeup Yoon

**Affiliations:** 1 Department of Health Sciences and Technology, Samsung Advanced Institute for Health Sciences and Technology, Sungkyunkwan University, Seoul 06351, Korea; minbk1991@gmail.com (B.M.); unohrkim@gmail.com (H.K.); 2 Institute for Refractory Cancer Research, Research Institute for Future Medicine, Samsung Medical Center, Seoul 06351, Korea; ymy1103@gmail.com (M.Y.); wm5016@naver.com (M.C.); 3 Institute for Future Medicine, Samsung Medical Center, Seoul 06351, Korea; 4 Department of Neurosurgery, Samsung Medical Center, Sungkyunkwan University School of Medicine, Seoul 06351, Korea; 5 Department of Biopharmaceutical Convergence, Sungkyunkwan University, Seoul 06351, Korea

**Keywords:** phage display, cell-based panning, semi-automated cell panning, FGFR3-specific antibody

## Abstract

Phage display technology is a widely used practical tool for isolating binding molecules against the desired targets in phage libraries. In the case of targeting the membrane protein with its natural conformation, conventional bio-panning has limitations on the efficient screening of the functionally relevant antibodies. To enrich the single-chain variable fragment (scFv) pools for recognizing the natural conformation of the membrane targets, the conventional bio-panning and screening process was modified to include the semi-automated cell panning protocol. Using FGFR3-overexpressing patient-derived cancer cells, biotin-X-DHPE was introduced and coupled to Streptavidin-coated magnetic beads for use in the solution-phage bio-panning procedure. The resulting clones of scFv were compared to the diversity of the binding region, especially on CDR-H3. The clones enriched further by cell-based panning procedure possessed a similar binding site and the CDR-H3 loop structure. The resulting antibodies inhibited cell growth and induced target degradation. This process may be a useful tool for screening biologically related antibodies that recognize natural conformational structure on cell membrane protein. Furthermore, cell-based panning has the potential to further expand to a high-throughput screening (HTS) system and automation process.

## 1. Introduction

Phage display, first described in 1985, is a practical tool for displaying proteins or peptides of interest in bacteriophage through fusion with viral envelope proteins [1,2,3]. Phage libraries are used to select and isolate binding molecules with high affinity for the target antigen with applications in monoclonal antibody (mAb) discovery, affinity maturation, and humanization [4,5]. Bio-panning for affinity selection has been used to isolate target protein-binding molecules from phage libraries [1,6,7]. The bio-panning procedure includes four major steps for phage selection: (i) Incubating and binding the phage library with the desired target; (ii) washing for non-binding and non-specific phage removal; (iii) eluting the specific phage binders; (iv) amplifying for eluted phages through Escherichia coli re-infection [8,9,10,11].

Conventional bio-panning has been based on various selection methods such as solid-phase for immobilized purified antigen, solution-phase using biotinylated antigen, and whole cell panning (WCP) [2,12]. The solid-phase selection is a fairly straightforward technique; however, the antigen must be presented in the correct conformation. Otherwise, the binders could recognize the epitopes that are naturally masked in the native form [12,13]. WCP was applied to isolated phage binders using cells intact with cell membrane proteins such as G-protein-coupled receptors, ligand-gate ion channels, receptor tyrosine kinases, and immunoglobulin-like receptors [3]. Although WCP may select non-specific phage binders to off-target cell surface protein, it could potentially enrich the phage binders recognizing the naturally exposed epitope on the cell surface [12,14,15].

We utilized the cell-based panning process in addition to the conventional bio-panning to enrich the phage binders specific for FGFR3 with the appropriate biological functions. The semi-automated cell panning method was optimized to maintain the advantages of the WCP by labeling a cell membrane-like substance (biotin-X-DHPE) and Streptavidin-coated magnetic beads without damaging the membrane proteins of intact cells [16]. Using FGFR3-overexpressing patients-derived cells (glioblastoma, GBM), biotin-X-DHPE and Streptavidin-coated magnetic beads were coupled to use in the solution-phage bio-panning procedure. It was applied to an automatic mechanical system using a magnetic particle processor (Thermo Fisher Scientific, KingFisher™ Flex, Waltham, MA, USA) to efficiently perform and reduce the screening time for WCP [17,18,19].

Fibroblast growth factors (FGFs) and their receptor (FGFR) signaling have functional roles in the regulation of cell proliferation, differentiation, and apoptosis. FGFR3 consists of an extracellular domain containing three immunoglobulin-like (Ig-like) domains (D1–D3), a transmembrane domain, and two intracellular tyrosine kinase domains. FGFR3 has two main splice variants FGFR3-IIIb and -IIIc. Aberrantly activated and/or overexpressed FGFR3 has been implicated in various human malignancies [20,21,22,23,24,25]. Several therapeutic antibodies targeting FGFR3 have been used in clinical development, such as monoclonal antibodies (Vofatamab, Rainier Therapeutics, San Leandro, CA, USA) or antibody-drug conjugates (LY3076226, Eil Lilly) [26,27,28].

In this study, we successfully selected FGFR3 antibodies with relevant biological function from a synthetic human single-chain variable fragment (scFv) phage library through the introduction of semi-automated cell panning. Sequence analysis and Fv modeling of the selected scFvs were performed, and sequence and structural similarity of CDR-H3 were analyzed using multiple alignments. In individual scFv, the respective IgG antibodies were generated based on scFvs and demonstrated the specific binding properties and biological functions such as inhibition of cell viability and target degradation. The introduction of a semi-automated cell panning process may be an efficient tool for selecting antibodies with functionality and specificity for cell membrane proteins in the generation of antibodies and antibody engineering.

## 2. Results

### 2.1. Introduction Strategy of Semi-Automated Cell Panning in Addition to Conventional Bio-Panning

**Figure 1:**
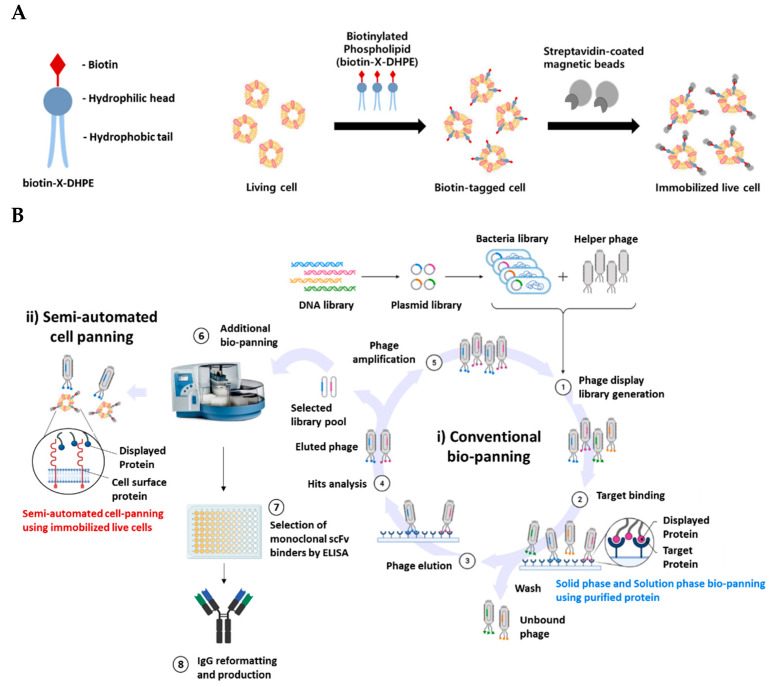
(**A**) Scheme for attachment of magnetic beads to live cells for applying bio-panning. Biotin-X-DHPE, a biotin tagged material, is a cell membrane-like substance composed of a hydrophilic head and a hydrophobic tail. Biotin-X-DHPE reacted with Streptavidin-coated magnetic beads to form a complex that is attached to the surface of living cells. (**B**) Bio-panning workflow combined with semi-automated cell panning. Binding to the rescued phage pool and target protein (Step 1–2). Wash to remove non-binding phage and then elute and amplify phage that binds to target protein (Step 3–5). Phage pool that recognizes naive target protein is isolated and screened by performing semi-automated cell panning on target-expressing cells (Step 6–8). Images created using BioRender.

We immobilized live cells using the complex of biotin-X-DHPE and Streptavidin-coated magnetic beads. This implied that the phospholipid-magnetic beads complex-labeled cells can be applied to magnetic beads-based bio-panning, as well as to the automated magnetic particle processor for semi-automated cell panning (Figure 1A). To efficiently enrich scFv binders, a semi-automated cell panning process was combined with conventional bio-panning. The scFv binders recognize the natural conformational structure expressed on the cell surface. First, 3–5 rounds of conventional bio-panning such as solid phase (e.g., immobilized antigen coating) or solution phase (e.g., magnetic beads-based with biotinylated antigen) were performed to enrich the phage pool bound to the purified protein. Next, the isolated phage pools underwent semi-automated cell panning to finally isolate scFv binders that recognize the cell surface 3D structure (Figure 1B).

To efficiently select clones that recognize both the purified target protein and cell surface membrane protein from a synthetic human scFv phage library, semi-automated cell panning was performed following conventional bio-panning [29]. In the case of conventional bio-panning such as bio-panning using purified protein or WCP, since clones are initially selected by a single method, it is difficult to select clones with desired binding characteristics using the bio-panning procedure (time of bio-panning: >8 weeks including repeated experiments). Conversely, in the case of semi-automated cell panning, clones that show biologically relevant properties and binding properties during the bio-panning procedure are enriched. It minimizes repetition of the bio-panning cycle in the in vitro binding test step (e.g., affinity or cell binding assay by ELISA) because unwanted clones are not selected and hence, the process is less time-consuming. Therefore, the required time and the burden of economic costs efficiently decrease because the bio-panning step is not re-performed (time of bio-panning: 3–4 weeks).

### 2.2. Optimal Immobilization Condition of Cell for Applying Semi-Automated Cell Panning

**Figure 2:**
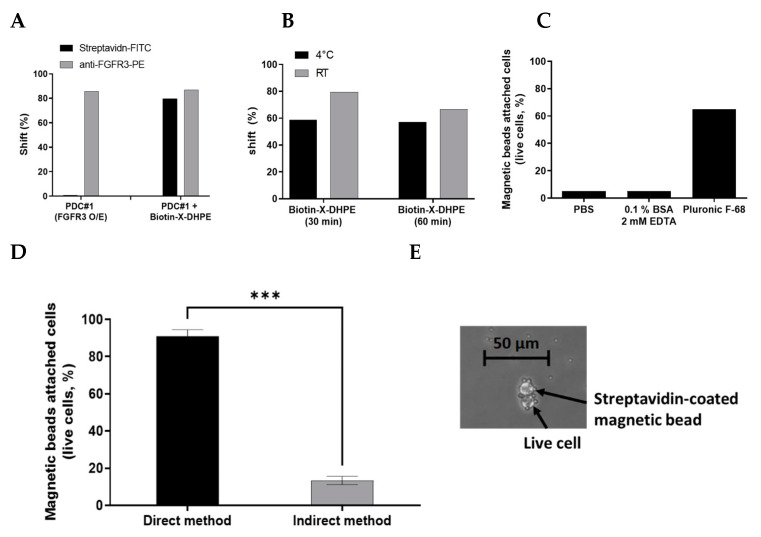
(**A**) Following biotin-X-DHPE labeling on FGFR3-overexpressing cells (PDC #1), FGFR3 expression levels on the cell surface were analyzed by FACS analysis. Biotin-X-DHPE was detected using Streptavidin-FITC, and FGFR3 was detected using PE-direct conjugated antibody. (**B**) The optimal reaction temperature and time for labeling the living cells with biotin-X-DHPE were analyzed by FACS analysis. (**C**) Optimal reaction buffer analysis for attaching magnetic beads on cells. (**D**) The capture yield of live cells attached with magnetic beads was compared using both direct and indirect method through cell counting and microscopic observation. *** *p* < 0.001 paired *T* test. (**E**) Microscopic observation of live cells attached with magnetic beads (magnification 400×).

To confirm whether biotin-X-DHPE affects the expression level or cell viability of the target protein on the cell surface after conjugation to the cell membrane, the conjugation of biotin-X-DHPE to cell membranes and attachment into living cells were assessed by FACS analysis. Biotin-X-DHPE was detected using Streptavidin-FITC (SA-FITC), FGFR3 was detected using anti-FGFR3-PE-conjugated control antibody, and histogram (shift, %) of FACS analysis was compared. The percent change in FITC intensity was approximately more than 80% after incubation with biotin-X-DHPE while that of PE was not changed, indicating that most of the cells were efficiently labeled with biotin-X-DHPE with no expressional change of FGFR3. Additionally, the FGFR3 expression level before and after treatment with biotin-X-DHPE remained unaltered, irrespective of the FGFR3 expression level on the cell surface (Figure 2A). On comparing the reaction time with the temperature at which biotin-X-DHPE is most effectively inserted into living cells, it was confirmed that more than 80% of biotin-X-DHPE was most attached to the cell surface under the reaction conditions of 30 min at room temperature using FACS analysis (Figure 2B). The labeling buffer was optimized by comparing various reaction buffers such as PBS (pH 7.4), 2 mM EDTA (0.1% BSA), and Pluronic F-68 buffer during cell labeling. Pluronic F-68 buffer was found to ensure more efficient cell labeling compared to other buffers under the same conditions (magnetic beads attached cells at more than 65%) (Figure 2C). Since it is a nonionic surfactant, it is considered useful for reducing the formation of bubbles that occur during agitation and incubation and for reducing the adhesion of cells to materials such as glass.

There are direct or indirect methods for labeling cells with biotin-X-DHPE and Streptavidin-coated magnetic beads (Figure S1). In the direct method, biotin-X-DHPE and Streptavidin-coated magnetic beads are incubated prior to forming a complex, and then the complex is attached to the cell. In the indirect method, biotin-X-DHPE is first attached to the living cells and then further conjugated with the beads. When comparing the two methods, the direct method showed a significantly higher labeling efficiency than the indirect method. The direct method showed approximately 85% or more labeled yield, while the indirect method showed less than 20% yield (Figure 2D). Furthermore, it was confirmed through microscopic observation that a complex consisting of biotin-X-DHPE and Streptavidin-coated magnetic beads was attached to the living cells (Figure 2E). The bio-panning scheme was modified from the conventional semi-automation panning to accommodate the semi-automated cell panning procedure. The dedicated software protocol for the optimal semi-automated cell panning process was modified, and the appropriate detailed scheme is included in Figure S2.

### 2.3. Isolation of FGFR3-Specific Clones through Introduction of Semi-Automated Cell Panning

We performed two conventional bio-panning: (1) Solution phase selection with a biotinylated antigen using semi-automated bio-panning; (2) solid phase selection with immobilization antigen coating using purified human FGFR3-IIIc. In the solution phase, semi-automated bio-panning using biotinylated human FGFR3-IIIc was performed using a magnetic particle processor. Using an automated processor and the included driving software, biotinylated FGFR3-IIIc and Streptavidin-coated magnetic beads were combined and then incubated with the rescued phage pool. Next, to collect FGFR3-IIIc-specific phage pools, the solution was washed and eluted at the designated plate. By performing five rounds of bio-panning, the scFv binders specific for human FGFR3-IIIc were amplified in the solution phase. In the solid phase, the process of amplifying the FGFR3-specific scFv binders was performed four times by immobilizing human FGFR3-IIIc to the immuno-tube and then treating the phage pool. The output to input phage titer ratio was calculated for each bio-panning round to confirm the amplification of the FGFR3-specific scFv binder pool. The ratio was improved after five rounds or four rounds of bio-panning compared to that after the first round (Table 1). Using conventional panning, 376 and 658 clones were screened and then 8 and 11 clones were isolated in the solution and solid phase, respectively, (cut-off > 2, relative O.D) with different sequences via ELISA screening analysis (Table 2).

To select clones, which recognize the surface FGFR3 protein of cells (PDC #1), semi-automated cell panning was performed for each phage pool that specifically binds to FGFR3-IIIc protein purified by two conventional bio-panning method, and input and output phages were titrated (Table 1).

**Figure 3:**
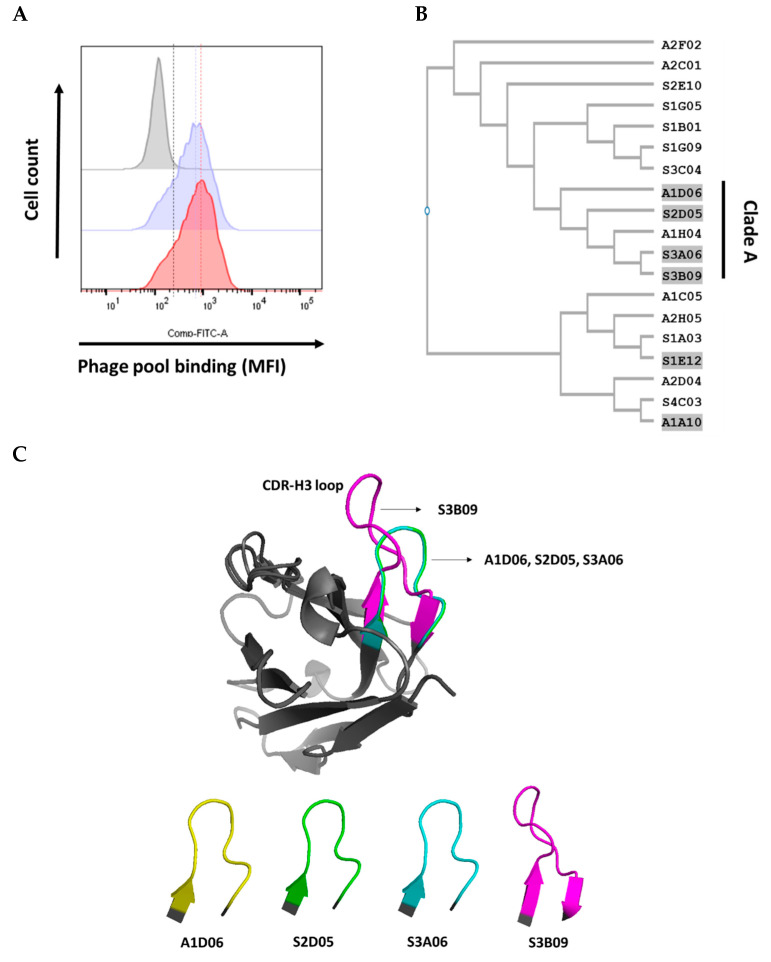
(**A**) Phage pool binding to FGFR3-overexpressing cells in a flow cytometer; comparison of cell surface binding between amplified phage pools obtained using conventional bio-panning (blue histogram) only or with the introduction of cell panning (red histogram). Comparison of CDR-H3 sequence similarity of isolated antibodies through cell panning introduction. (**B**) Phylogenetic tree of similar sequences built on the basis of CDR-H3 (Kabat numbering) using CLUSTAL W multiple sequence alignment programs. Each phylogenetic tree was analyzed for 13 clones selected through conventional bio-panning only and 6 clones selected through introduction of cell panning. The A1D06, S2D05, S3A06, and S3B09 clones were grouped into “Clade A”. (**C**) Comparison of CDR-H3 loop structure alignment using POSA analysis (interactive multiple protein structure alignment). The CDR-H3 of the A1D06 (yellow), S2D05 (green), S3A06 (cyan), and S3B09 (magenta) clones of Clade A were aligned based on the VH region in the Fv modeling annotation. CDR-H3 loop structure and length are different for each clone (gray, framework).

We compared the binding efficiency of each phage pool rescued from conventional bio-panning and additional semi-automated cell panning to FGFR3-overexpressing cells (PDC #1). Each output phage pool was collected through PEG precipitation and titration, and the same amount of phage particles was bound to FGFR3-overexpressing cells, and then the fluorescence intensity was compared using flow cytometry. The output phage pool in which semi-automated cell panning was further introduced had partially enhanced the selectivity for the FGFR3-overexpressing cells as compared with the output phage pool in which conventional bio-panning was performed (Figure 3A).

After the introduction of semi-automated cell panning procedure, we screened 188 and 372 individual clones and isolated 2 and 4 clones each (cut-off > 2, relative O.D) with different sequences via ELISA screening analysis (Table 2). The sequences of the six clones selected by introduction of semi-automated cell panning are included in the 19 clones of conventional bio-panning sequences. Clones with the characteristics of binding to the surface of FGFR3-overexpressing cells were enriched in the phage pool selectively amplified for purified FGFR3-IIIc by conventional bio-panning.

### 2.4. Complementarity-Determining Regions of the Heavy Chain (CDR-H3) Sequence Analysis and Structure Homology Alignment Using Variable Fragment (Fv) Modeling

The sequences of CDR-H3 (Kabat numbering) were compared in the six CDR regions (CDR-L1, L2, L3, H1, H2, and H3) of the antibody [30,31,32]. The CDR-H3 region of an antibody has the most sequence and structural diversity and is known to play the most important role in antigen-binding specificity among the six CDRs [33,34,35,36]. Moreover, the synthetic human scFv library used in this study also has the most diverse sequence and structural features of CDR-H3 [29].

The nucleotide and amino acid sequence of phagemid vector for scFvs (VH-linker-VL) were analyzed using 19 clones of different sequences isolated by conventional bio-panning and 6 clones (included in 19 sequences) selected by semi-automated cell panning (cell panning) established in this study. The CDR-H3 sequences of 19 different clones selected by bio-panning were confirmed to have various lengths and amino acid configurations. Six clones (A1D06, S2D05, S3A06, S3B09, S1E12, and A1A10) selected by cell panning were marked with gray highlights (Figure 3B). The CDR-H3 sequences of the six clones were compared for similarity through phylogenetic tree analysis using a Clustal W (multiple sequence alignment programs) tool. Clones A1D06, S2D05, S3A06, and S3B09 (Clade A) were significantly similar in sequence and length to CDR-H3, but S1E12 and A1A10 showed differences (Figure 3B). To compare the CDR-H3 structural similarity between the selected six clones, Fv modeling was first performed using the SAbPred (a structure-based antibody prediction server) tool (Figure S3) [37]. The structure alignment of the CDR-H3 loop was performed using the POSA (partial order structure alignment) tool for the whole variable heavy chain (VH), and the structural similarity of CDR-H3 was analyzed using a flexible multiple structure alignment approach for Clade A [38,39]. The average of root-mean-square deviation (RMSD) in the VH region (tertiary structure) between each clone in Clade A was less than 1 Å, which is the general criterion considered for significant similarity (Figure 3C) [40]. Therefore, clones of Clade A with sequence and structurally similarity to CDR-H3 were considered because they have similar binding properties against FGFR3. However, the overall CDR-H3 sequence and structural similarity pattern of Clade A may not be completely identical. It has a limitation in that there may be ambiguous contradictions due to the difference between the methodology of the two alignments.

### 2.5. Generation of Anti-FGFR3 Antibodies (IgG) and Analysis of Physicochemical Properties

The VH and VL sequences of the anti-FGFR3 scFv binders selected using cell panning were analyzed, and each sequence was cloned into heavy (Immunoglobulin G1, IgG1) and light chain (lambda) expression vectors for reformatting IgG (Figure S4). Anti-FGFR3 antibody clones were produced by co-transfection of heavy and light chain vectors using the Expi293 expression system and highly purified clones were obtained using an affinity chromatography column. The production yield of each clone was as follows: A1D06, 20 mg/L; S2D05, 120 mg/L; S3A06, 161 mg/L; S3B09, 100 mg/L; S1E12, 180 mg/L; A1A10, 38 mg/L after 6 days of incubation.

**Figure 4:**
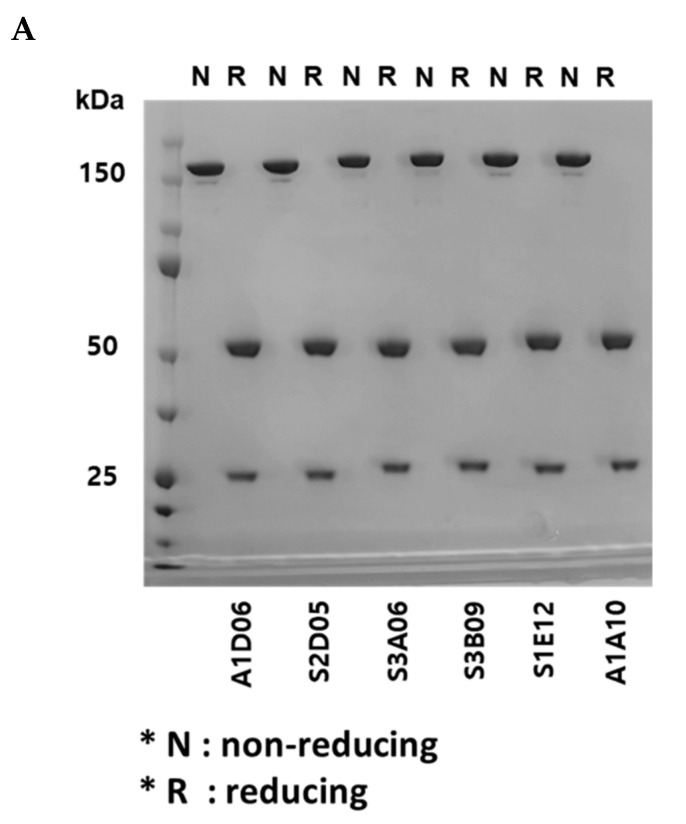

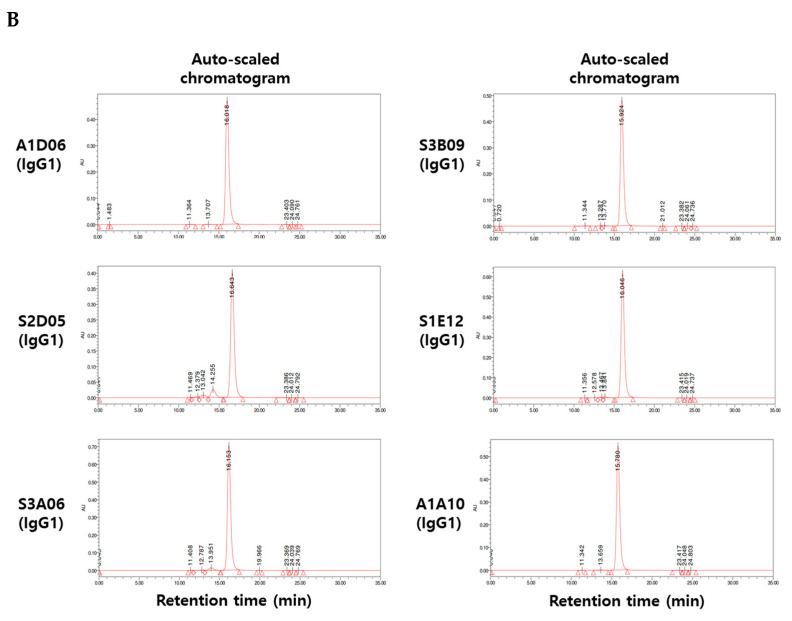
Physicochemical property analysis of anti-FGFR3 antibodies derived from cell panning. (**A**) Sodium dodecyl sulfate polyacrylamide gel electrophoresis (SDS-PAGE) analysis of purified antibodies in non-reducing and reducing conditions. In the non-reducing condition, 150 kDa band indicates the whole IgG, and in the reducing condition, 25 kDa and 50 kDa indicate the light chain and heavy chain of the antibody, respectively. (**B**) The purity of the anti-FGFR3 antibodies were analyzed using size exclusion-high performance liquid chromatography (SEC-HPLC). All anti-FGFR3 antibodies showed more than 95% purity of monomer.

Using sodium dodecyl sulfate polyacrylamide gel electrophoresis (SDS-PAGE) analysis, the assembly of heavy and light chains was confirmed and the molecular weight of whole IgG was analyzed. Under non-reducing conditions, all clones were observed to have a molecular weight of approximately 150 kDa, whereas under reducing conditions, heavy and light chains were observed to have molecular weights of approximately 50 kDa and 25 kDa, respectively (Figure 4A). By using size exclusion-high-performance liquid chromatography (SEC-HPLC), all clones were analyzed to confirm their purity, and their physical properties showed to be 95% or more (Figure 4B). It indicates that there was no loss of monomer such as through fragmentation (i.e., low-molecular-weight, LMW species) or aggregation (i.e., high-molecular-weight, HMW species).

### 2.6. Binding Properties of Anti-FGFR3 Antibodies

**Figure 5:**
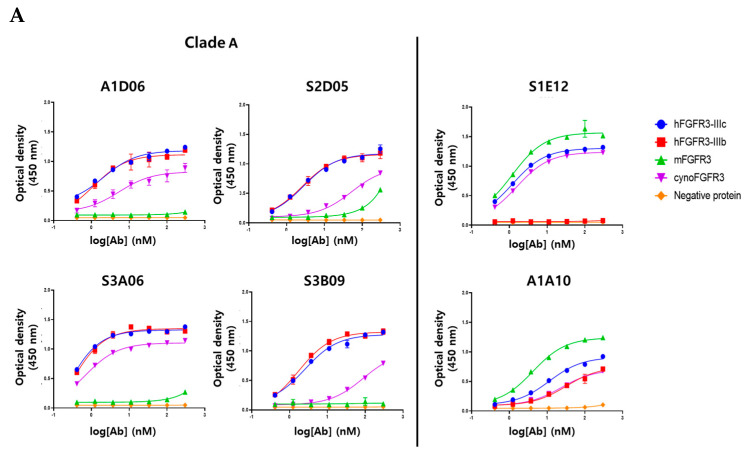

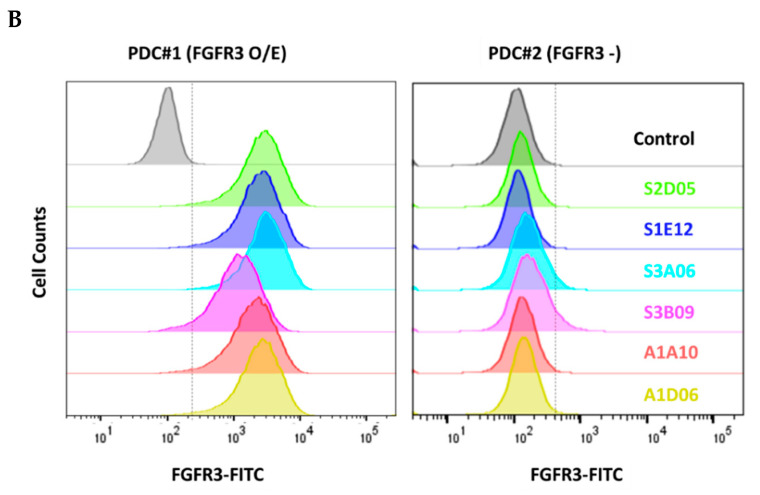
Binding characterization analysis of anti-FGFR3 antibody derived following the introduction of semi-automated bio-panning. (**A**) The binding specificity of selected FGFR3 antibodies to FGFR3 isoforms and interspecies was evaluated using ELISA. (**B**) The binding ability of FGFR3 antibodies to FGFR3-overexpressing cells (PDC#1) and FGFR3 negative cells (PDC#2) was determined using flow cytometry.

To verify the target-specific binding ability of the anti-FGFR3 antibodies, purified human FGFR3 isoform (FGFR3-IIIb, -IIIc) and several species of FGFR3 (mouse FGFR3-IIIc, cynomolgus monkey FGFR3-IIIc) were used in an ELISA assay. All clones showed specific binding for the purified human FGFR3-IIIc antigen, which were used for bio-panning, dependent on their concentration, but not to the negative protein (Fc-tagged). The six clones were considered to have apparent specificity for purified human FGFR3-IIIc (Figure 5A). Clade A (A1D06, S2D05, S3A06, and S3B09 clones), clustered based on the similarity of CDR-H3 sequences, was confirmed to have cross-reactivity between human FGFR3-IIIb and cynomolgus FGFR3-IIIc, but did not show specificity for mouse FGFR3-IIIc. The clone S1E12 was not specific to human FGFR3-IIIb, but it had apparent binding specificity for mouse FGFR3-IIIc and cynomolgus FGFR3-IIIc. It was hypothesized that this clone has a binding epitope for the IIIc region of the FGFR3 extracellular domain. The first half of the Ig III domain of FGFR3 is encoded by the invariant exon (IIIa), and the other half is the region where the variant occurs by splicing to IIIb or IIIc [24]. The clone A1A10 was estimated to have a homologous region of human (IIIb and IIIc), mouse, and cynomolgus FGFR3 as an epitope, as it binds to the other FGFR3 proteins as well as human FGFR-IIIc. Clones of Clade A were considered to have similar binding patterns to the human FGFR3 isoform or interspecies FGFR3 due to sequence and structural similarities of CDR-H3.

To accurately measure the binding kinetics (KD value) of anti-FGRF3 clones, we determined the affinity of the antibody to each purified protein based on surface plasmon resonance (SPR) analysis. Each clone showed the same binding pattern as the ELISA assay for isotype and interspecies of FGFR3 proteins (Figure S5). Clade A bound to hFGFR3-IIIc, hFGFR3-IIIb, and cynoFGFR3-IIIc, but did not bind to mFGFR3-IIIc. Clone S1E12 showed specific affinity for FGFR3-IIIc regardless of species and did not bind to FGFR3-IIIb. Clone A1A10 showed specificity for all types of FGFR3 proteins. The affinity of each clone for the FGFR3-purified protein is distributed from about ten nanomolar to sub-nanomolar, and it is considered that could be applied as a therapeutics (Table 3).

We verified that the selected clones bind to the FGFR3 expressed on cell surface using FACS analysis. All clones showed specific binding to FGFR3-overexpressing cell (PDC #1) and did not bind to FGFR3-negative cell (PDC #2) (Figure 5B). Thus, the clones selected through the introduction of cell panning were proved to have specific binding affinity for the purified FGFR3 protein as well as the natural FGFR3 tertiary structure on the cell surface membrane.

### 2.7. Biological Function Analysis of Anti-FGFR3 Antibodies to FGFR3-Overexpressing Cells

**Figure 6:**
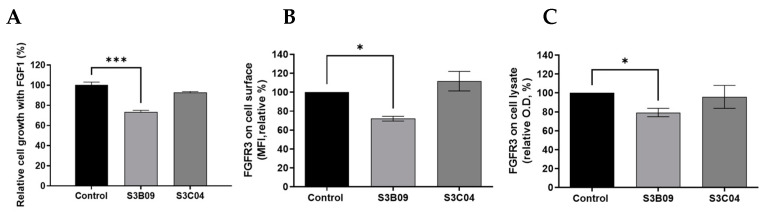
Biological functional assay of anti-FGFR3 antibodies. Inhibitory effect of clone S3B09 (Clade A) and S3C04 selected from conventional bio-panning on proliferation of FGFR3-overexpressing cells (PDC #1) was evaluated. Cells were cultured in the presence of 10 ng/mL FGF1 plus 10 μg/mL heparin sulfate or with S3B09 or S3C04 antibodies. Relative cell growth was evaluated through (**A**) cell viability after 96 h incubation with antibodies. Data represent mean ± SD; ***, *p* < 0.001 using one-way ANOVA. (**B**) Cell surface FGFR3 degradation assay. Individual antibodies were treated on FGFR3-overexpressing cells (PDC #1) and incubated for 1 h at 37 °C, and FGFR3 expression on the cell surface was detected through FACS analysis. (**C**) Total FGFR3 degradation assay. After the FGFR3-overexpressing cells (PDC #1) were treated with the antibody, total FGFR3 contained in the cell lysate was detected through sandwich ELISA. Data represent mean ± SD; *, *p* < 0.03 using one-way ANOVA.

To compare biological functions, in vitro functional assays for S3B09 of Clade A, which is the representative clone selected by cell panning, and S3C04 selected by conventional bio-panning were performed on FGFR3-overexpressing PDCs. The cell growth inhibition of anti-FGFR3 antibodies (S3B09, S3C04) was evaluated for FGFR3-overexpressing cells incubated with FGF1 ligand. To accurately analyze the anti-proliferation effect of the antibody, cell viability was assessed. After 96 h of antibody treatment, it was confirmed that the S3B09 selected by cell panning inhibited the tumor growth by approximately 30% (Figure 6A). The S3C04 selected by conventional bio-panning inhibited the tumor growth by less than 10%. It means that S3B09 antibody significantly inhibits the cell growth compared to control IgG and S3C04 against FGFR3-overexpressing PDCs.

To confirm the mechanism of inhibition of S3B09 on FGFR3-overexpressing cells, the induction of FGFR3 degradation by the antibody was verified. Changes in FGFR3 expression level on the cell surface was analyzed using flow cytometry and the change in total FGFR3 protein level was analyzed using sandwich ELISA. After incubating the S3B09 or S3C04 with the FGFR3-overexpressing cell line for 1 h, changes in the FGFR3 expression level on the surface of living cells were confirmed. S3B09 decreased the FGFR3 expression level by 28% compared to control IgG; however, S3C04 did not affect the FGFR3 expression level (Figure 6B). Additionally, after treatment with S3B09 or S3C04, the total FGFR3 protein level was evaluated using the lysate of the FGFR3-overexpressing cells, and the total FGFR3 protein level decreased by approximately 20% when treated with S3B09 (Figure 6C). These results suggest that S3B09 antibody selected by the introduction of cell panning effectively inhibits the growth of FGFR3-overexpressing cells through FGFR3 degradation.

## 3. Discussion

In phage display with WCP, the binders are separated in a state where the membrane protein is expressed in a natural tertiary structure to recognize the epitope of the naturally exposed region [3,10,13]. Therefore, WCP could represent a suitable strategy to obtain antibodies that are conformational-specific for membrane protein such as G protein-coupled receptors, ligand-gate ion channels, receptor tyrosine kinases, and immunoglobulin-like receptors, etc. [3,41,42,43,44]. Various cell-based panning strategies have been studied and optimized over a long period of time such as shadow-stick selection technique, FACS sorting technique, and bio-panning and rapid analysis of selective interactive ligands (BRASIL) [3,45,46,47,48]. However, technical optimization is required to reduce the non-specific binders to the common cell surface proteins or the irrelevant proteins and to improve the time-consuming process using the intact cells [12,14].

To efficiently separate the phage binder that binds to the cell surface membrane protein, we designed a screening process that enriches the binder using conventional bio-panning along with a semi-automated cell panning process. We optimized a method of labeling cells with biotin-X-DHPE (biotinylated phospholipid), the substance with a biotin tag on a cell membrane-like structure, to apply living cells to magnetic beads-based panning. Since this substance has a hydrophilic head and a hydrophobic tail similar to the structure of cell membrane phospholipids, it can be easily immobilized to the cell membrane while minimizing cell damage. Since the biotin tag has a strong affinity for Streptavidin, Streptavidin-coated magnetic beads can be efficiently used with cells during the bio-panning procedure. The PDCs were conjugated with biotin-X-DHPE and attached to the Streptavidin-coated magnetic beads under optimized conditions to maintain viable cells with the natural conformation of the membrane protein [16]. Additionally, we implemented this method using an automatic instrument as the magnetic particle processor owing to its advantages in terms of time and labor required to efficiently isolate binders [17,18,19]. Furthermore, it may be applicable for high-throughput screening using multiple libraries, and binders can be selected simultaneously using various cells such as PDCs and cancer cell lines.

Abnormal FGFR3 signaling due to overexpression and/or mutation induces tumor proliferation and metastasis in multiple tumors [20,21,22,23,24,25]. Several antibody therapeutics targeting FGFR3 have been developed such as mAb, Vofatamab (Rainier Therapeutics) currently undergoing phase 1/2(b) clinical trial and Antibody-drug conjugate, LY3076226 (Eli Lilly) currently undergoing phase 1 clinical trials [49,50,51]. In our laboratory, we tried to screen the specific and functional antibody targeting FGFR3; however, it was difficult to isolate the binders with degradation and/or internalization properties (data not shown).

In this study, we enriched the functional antibodies through the additional semi-automated cell panning process using PDCs (glioblastoma, GBM) with a high level of FGFR3 expression. The 19 individual clones were isolated using the 1034 clones obtained from four or five rounds of the conventional bio-panning, and the 6 individual clones using 560 clones obtained from the additional semi-automated cell panning. The IgGs reformatting and production were performed on isolated clones, and target specificity was confirmed through affinity ELISA analysis and SPR analysis for the purified FGFR3 protein. Through the evaluation of the functional analysis, the final 4 candidates were selected. The semi-automated cell panning showed the enrichment of the desired 4 candidates from the 6 clones in comparison to the 4 of 19 clones obtained from conventional bio-panning. All of the 4 clones showed binding specificity for FGFR3-overexpressing cells, and non-specific binding or unwanted binding patterns were not observed. We performed cell proliferation and target degradation assays to analyze the biological function against FGFR3-overexpressing cells. The candidate clone obtained through the introduction of semi-automated cell panning was shown to inhibit tumor growth and degrade FGFR3 on the cell surface; however, the remaining clones (15 clones of 19 clones) obtained through conventional bio-panning alone showed poor biological-related function against the FGFR3-overexpressing cells.

In summary, we optimized the semi-automated cell panning method and then introduced an additional process to enrich binders that have specific binding properties with natural conformation of cell surface protein. It was applied to efficiently select FGFR3-specific antibodies that have binding specificity and biologically relevant function. Finally, we selected a clone that showed anti-tumor effects and FGFR3 degradation against FGFR3-overexpressing cells. This method may be an efficient screening tool for isolating clones that recognize membrane protein structure and have biological functions in antibody discovery through phage display. Furthermore, it has the potential to extend cell panning procedure to HTS systems and fully automated systems.

## 4. Materials and Methods

### 4.1. Immobilization of Cells Using Biotin-X-DHPE and Coated Magnetic Beads

PDCs (Glioblastoma, GBM) were dispensed into the tubes at a density of 5.0 × 10^5^ cells/mL. The cells were centrifuged at 1500 rpm for 3 min with 1% FBS (in PBS, pH 7.4, Thermo Fisher Scientific, Waltham, MA, USA) and washed twice to remove the supernatant. Fluorescein DHPE (N-(Fluorescein-5-Thiocarbamoyl)-1,2-Dihexadecanoyl-sn-Glycero-3-Phosphoethanolamine, Triethylammonium Salt, Invitrogen, F362, Carlsbad, CA, USA) or biotin-X-DHPE (1,2-dihexadecanoyl-sn-glycero-3-phosphoethanolamine, Invitrogen, B1550, Carlsbad, CA, USA), a cell membrane-like substance, was incubated with the cells to attach magnetic beads to the surface of living cells in a rotator at room temperature or 37 °C for 30 or 60 min. Cells labeled with biotin-X-DHPE were further incubated with Streptavidin-FITC (Invitrogen, SA1001, Carlsbad, CA, USA), which has biotin-specific binding force, at 4 °C for 1 h and washed twice with 1% FBS wash buffer. Flow cytometry (BD FACSAria™ III Cell Sorter) was used to analyze whether biotin-X-DHPE was fused to the cell surface. To attach magnetic beads to cells labeled with biotin-X-DHPE, 2 mg of Dynabeads™ M-280 Streptavidin (Invitrogen, 11206D, Carlsbad, CA, USA) was washed twice in PBS using a magnetic separation rack and incubated with the cells at room temperature for 1 h. After the reaction, the cells were washed through a magnetic separation rack and observed using a microscope to confirm whether the cells were attached to the biotin-X-DHPE and the magnetic beads complex.

### 4.2. Optimization of Cell Immobilization Conditions

The direct beads mounting method is as follows: 3 μg of biotin-X-DHPE and 200 μg of Dynabeads™ M-280 Streptavidin were first incubated at room temperature for 30 min and then isolated using a magnetic separation rack to form biotin-X-DHPE-magnetic beads complex. Then, the complex was incubated with 1.0 × 10^6^ to 5.0 × 10^6^ living cells in a rotor at room temperature for 1 h and then the washing was performed to isolate the immobilized cells.

The indirect beads mounting method is as follows: 3 μg of biotin-X-DHPE was incubated with living cells using a rotor at room temperature for 30 min and then washed. The pre-washed Dynabeads™ M-280 Streptavidin was then incubated with cells labeled with biotin-X-DHPE to attach the beads to the cell surface.

To increase the efficiency of attachment between cells and magnetic beads, reaction conditions were optimized using PBS (pH 7.4), 2 mM EDTA/0.1% BSA, and 0.1% Pluronic F-68 (Gibco, 24040032, Carlsbad, CA, USA).

### 4.3. Bio-Panning Using Phage Display

Bio-panning for FGFR3-specific antibody screening was performed using the synthetic human scFv library [29]. Conventional bio-panning using the recombinant FGFR3-Fc tagged protein was performed using two methods: magnetic beads-based semi-automated bio-panning (solution phase) and immobilized antigen coating bio-panning (solid phase). In the case of the magnetic beads-based semi-automated bio-panning method, the recombinant FGFR3-Fc protein (R&D systems, 766-FR-050, Minneapolis, MN, USA) was biotinylated using Biotinylation kit (Abcam, ab201796, Cambridge, UK) and then a magnetic particle processor (Thermo Fisher Scientific, KingFisher™ Flex, Waltham, MA, USA) was used to perform semi-automated bio-panning. This automatic process was performed according to the protocol pre-designed through BindIt Software 3.3 and repeated five times to amplify the FGFR3-specific scFv binder pool. For the immobilized antigen coating method, the immuno-tube was coated with 3 μg each of recombinant FGFR3-Fc tagged protein and recombinant negative-Fc tagged protein. To remove the scFv binder that binds to the Fc tag through negative selection, the phage library pool was first incubated on an immuno-tube coated with a negative-Fc tagged protein. The supernatant was further incubated on the immuno-tube coated with the FGFR3-Fc tagged protein to obtain scFv binders specifically binding to FGFR3. The scFv binder pool that specifically binds to FGFR3 was amplified by performing four rounds of bio-panning.

### 4.4. Semi-Automated Cell Panning Using Immobilized Cells

Semi-automated cell panning was performed using beads-attached cells to rapidly isolate clones that bind to the surface of FGFR3-overexpressing cells from output of the previous two conventional bio-panning methods. First, the rescued phage pool was incubated with the FGFR3-negative cells and Streptavidin-coated magnetic beads for negative selection and only the supernatant was collected using a centrifuge. The recovered phage pool was placed in a 24-deep-well plate of KingFisher™ Flex, and approximately 1.0 × 10^6^ to 2.0 × 10^6^ of FGFR3-overexpressing cells labeled by biotin-X-DHPE and Streptavidin-coated magnetic beads were added into a dedicated plate (24-deep well) of an automated magnetic particle processor. Wash buffer (0.1% PBST), followed by elution buffer, was also added to the plates, and semi-automated cell panning was then performed using the pre-designed BindIt Software 3.3 protocol. From the phage pool recovered by semi-automated cell panning, clones which specifically bind to the FGFR3 protein but not the negative protein were isolated via affinity ELISA analysis for scFv screening.

### 4.5. 3D-Structure Modeling and Alignment Analysis of Anti-FGFR3 Antibodies

Structure-based prediction and design for antibody engineering were performed using a web-server called SAbPred [37]. This tool annotates antibody sequences, which are required for both heavy and light chains for modeling paired antibody, and automatically generates a homology model of the antibody Fv region. Six antibody sequences were selected for this study. The putative Fv model was annotated and refined into the region of CDR-H3 or not by using PyMOL.

Structural alignment was performed using a multi-protein structure alignment server called POSA, which uses multiple flexible structural alignment [38,39]. Fv models (PBD IDs) of six clones were submitted, and RMSD values and visualization modeling were verified based on the alignment results. Finally, annotation and refinement were performed using PyMoL.

### 4.6. IgG Reformatting and Production of Anti-FGFR3 Antibodies

The antibody variable region of isolated FGFR3-specific scFv was analyzed using phagemid vectors. The variable region sequences of heavy chain (IgG1) or light chain isolated from phagemid vectors were inserted into each mammalian expression vectors, respectively. Anti-FGFR3 antibodies were produced using the Expi293 transient mammalian expression system (Gibco, A14635, Carlsbad, CA, USA) through co-transfection of the above-mentioned vectors. Following transfection, the culture supernatant was purified using the ÄKTA protein purification system (GE Healthcare Life Sciences, Uppsala, Sweden) with HiTrap Mabselect SuRe (GE Healthcare Life Sciences, 11-0034-93, Uppsala, Sweden). After purification, enrichment was performed with Amicon^®^ Ultra Centrifugal Filter (Merck Millipore, MA, USA). The characteristics of the highly purified antibodies were analyzed using SDS-PAGE and SEC-HPLC.

### 4.7. ELISA Binding Assay

One microgram per milliliter of each human (FGFR3-IIIb and -IIIc) (R&D systems, 1264-FR-050, Minneapolis, MN, USA), mouse (R&D systems, 710-MF-050, Minneapolis, MN, USA), and cynomolgus FGFR3 (Sino Biological, 90313-C02H, Beijing, China) protein was coated on 96-well EIA/RIA plates (Costar, #3590, Corning, NY, USA) at 4 °C for over-night, respectively. The plates were blocked with 3% skim milk containing anti-FGFR3-antibodies and incubated for 1 h at room temperature. After washing with PBST (0.1%), the anti-human Fab antibody-conjugated horseradish peroxidase (HRP) (Thermo Scientific, 31482, Waltham, MA, USA) was added at a ratio of 1:3000 in 3% skim milk. Following the wash, the plate was treated with TMB solution (Thermo Scientific, N301, Waltham, MA, USA) as an HRP substrate, and the reaction was stopped with STOP solution (Cell Signaling Technology, #7002, Danvers, MA, USA). The absorbance for each well was detected at 450 nm wavelength with an Infinite^®^ M200 pro (Tecan, Männedorf, Switzerland).

### 4.8. Surface Plasmon Resonance Analysis

The binding affinity (KD values) of anti-FGFR3 antibodies was measured using Biacore 3000 (GE Healthcare Life Sciences, Uppsala, Sweden). The human (FGFR3-IIIb and -IIIc), mice, and cynomolgus FGFR3 proteins were immobilized on a Sensor Chip CM5 (GE Healthcare Life Sciences, 29149604, Uppsala, Sweden) with Amine coupling kit (GE Healthcare Life Sciences, BR100050, Uppsala, Sweden). The KD (Ka and Kd) value was assessed according to the concentration gradient of antibodies.

### 4.9. Cell Binding Analysis Using Flow Cytometry

Binding efficiency of phage pools to FGFR3-overexpressing cells was determined using flow cytometry (BD Biosciences, FACSAria III, Mountainview, CA, USA). The phage pools (approximately 5.0 × 10^12^ phage particles) precipitated with PEG were titrated in advance and blocked using 1% BSA at room temperature for 1 h. FGFR3-overexpressing cells at a density of approximately 3.0 × 10^5^ to 5.0 × 10^5^ cells/mL were washed in eBioscience™ Flow Cytometry Staining Buffer (Invitrogen, 00-4222-57, Carlsbad, CA, USA) and then incubated with pre-blocked phage pool at 4 °C for 1 h. After washing twice with a staining buffer, anti-HA tag antibody (Cell signaling technology, # 3724S, Danvers, MA, USA) was diluted 1:800 in a staining buffer and incubated at 4 °C for 1 h. After washing, goat anti-Rabbit IgG (H+L) cross-adsorbed secondary antibody conjugated with Alexa Fluor 488 (Invitrogen, A-11008, Carlsbad, CA, USA) was diluted 1:200 in a staining buffer and incubated for 30 min at 4 °C. The binding pattern of phage pools was analyzed using flow cytometry.

The cell-surface-binding efficiency of anti-FGFR3 antibodies was analyzed using flow cytometry. About 3.0 × 10^5^ to 5.0 × 10^5^ FGFR3-overexpressing or FGFR3 negative cells were incubated with anti-FGFR3 antibodies with 200 nM at 4 °C for 1 h. After washing twice with eBioscience™ Flow Cytometry Staining Buffer, the cells were stained with the goat anti-human IgG (H+L) cross-adsorbed secondary antibody conjugated with Alexa Fluor 488 (Invitrogen, A-11013, Carlsbad, CA, USA) diluted 1:200 in staining buffer at 4 °C for 30 min. Mean fluorescence intensity was analyzed by flow cytometry.

### 4.10. Assessment of Cell Growth

FGFR3-overexpressing cell were seeded with FGF1 ligand (10 ng/mL) plus heparin sulfate (10 μg/mL) in a 96-well plate at a density of 20,000 cells/well and incubated with 500 nM anti-FGFR3 antibodies for 96 h. The cell growth was assessed using an Ez-Cytox cell viability assay kit (DAEIL Lab, EZ-1000, Seoul, Korea) at an optical density of 450 nm using a microplate reader.

### 4.11. Target Degradation Assay

FGFR3 degradation of the selected antibody was confirmed by analyzing the FGFR3 on the cell surface following antibody treatment. FGFR3-overexpressing cells (3.0 × 10^5^) were incubated with 100 nM of anti-FGFR3-antibodies for 2 h at 37 °C. After washing with 1% FBS (pH 7.4, PBS), the cells were stained with human FGFR3 PE-conjugated antibody (R&D systems, FAB766P, Minneapolis, MN, USA) and the FGFR3 expression level on the cell surface was analyzed by FACS analysis.

FGFR3 degradation through total FGFR3 comparison of cells was determined using solid phase sandwich ELISA. Approximately 5000 to 10,000 FGFR3-overexpressing cells were cultivated on a 96-well plate, treated with 200 nM of anti-FGFR3 antibodies, and then incubated at 37 °C for 1 h. The cells were centrifuged to remove the supernatant and lysed with cOmplete™ Lysis-M buffer (Roche Diagnostics, 04719956001, Mannheim, Germany). The lysate was quantified and analyzed using sandwich ELISA at an optical density of 450 nm using human total FGFR3 DuoSet IC ELISA (R&D systems, DYC766-2, Minneapolis, MN, USA).

### 4.12. Statistical Data Analysis

All statistical analysis were performed using Graph Pad Prism software (GraphPad Software, Inc., La Jolla, CA, USA). One-way ANOVA using paired t-test was used to compare two or more data sets and the statistical significance was set at *p*-value < 0.03.

### 4.13. Ethical Statement

GBM specimens were obtained from patients undergoing surgery based on consent in accordance with the appropriate Institutional Review Boards. The study was approved by the Institutional Review Board of Samsung Medical Center (IRB No. 2010-04-004) and performed in accordance with the principles of the Declaration of Helsinki. Written informed consents were obtained.

## 5. Patent

A patent application has been filed in South Korea (application number: 10-2019-0125222).

## Figures and Tables

**Table 1:** Phage titer following conventional bio-panning and semi-automated bio-panning.

	Round	Input Titer (×10^10^ CFU)	Output Titer (×10^5^ CFU)	Overall % Yield (Output/Input × 100%)
Conventional panning	I	25.8	76.9	0.00298
: Solution phase selection using semi-automated bio-panning	II	5.5	1.46	0.00027
	III	23.9	238	0.00996
	IV	27.2	906	0.03331
	V	6.5	321	0.04938
Semi-automated cell panning	VI	18.3	561	0.03066
Conventional panning	I	100	1	0.00001
: Solid phase selection using immobilization antigen coating panning	II	2.5	0.05	0.00002
	III	2.9	0.12	0.000041
	IV	3	0.1	0.000033
Semi-automated cell panning	VI	49	13	0.000265

**Table 2:** Summary of sequenced clones obtained from conventional bio-panning and semi-automated bio-panning.

	Clones Screened	Different Sequences
Conventional panning : Solution phase selection using semi-automated bio-panning	376	8
Semi-automated cell panning *	188	2
Conventional panning : Solid phase selection using immobilization antigen coating panning	658	11
Semi-automated cell panning **	372	4

Semi-automated cell panning *: magnetic beads-based semi-automated panning (solution phase) + semi-automated cell panning; semi-automated cell panning **: immobilized antigen panning (solid phase) + semi-automated cell panning.

**Table 3:** Binding affinities (KD) of selected clones to human FGFR3 isotypes and interspecies FGFR3.

Antigen	Species	KD, nmol/L					
		Clade A					
		A1D06	S2D05	S3A06	S3B09	S1E12	A1A10
FGFR3-IIIc	Human	2.78	4.54	0.63	21.2	1.39	6.37
FGFR3-IIIb	Human	3.52	5.43	1.66	23.5	n.b.	15.8
FGFR3(IIIc)	Cynomolgus monkey	2.1	5.62	1.02	32.8	2.36	7.77
FGFR3 (IIIc)	Mouse	n.b.	n.b.	n.b.	n.b.	1.93	7.49

n.b.: non-binding.

## Data Availability

Not applicable.

## Supplementary Materials

The following are available online at https://www.mdpi.com/article/10.3390/ijms22126240/s1.
