# Supplementary material for: Semi-Automated Cell Panning for Efficient Isolation of FGFR3-Targeting Antibody
Source: Int J Mol Sci. 2021 Jun 9;22(12):6240. doi: 10.3390/ijms22126240 (PMC8229736; doi:10.3390/ijms22126240)

Supplementary Materials

Supplementary figure 1. Schematic representation of direct/indirect method of attaching beads to cells.

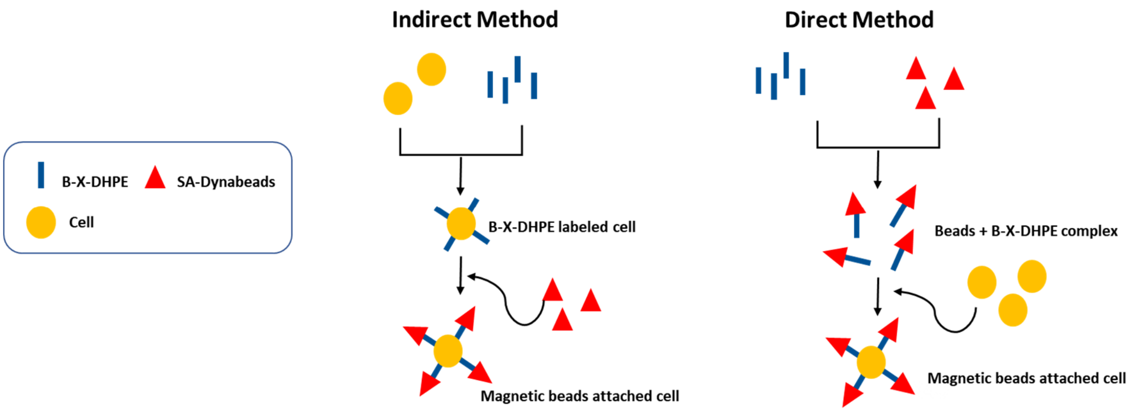

Supplementary figure 2. Scheme of the novel semi-automated cell panning technique with immobilized cells using a magnetic particle processor.

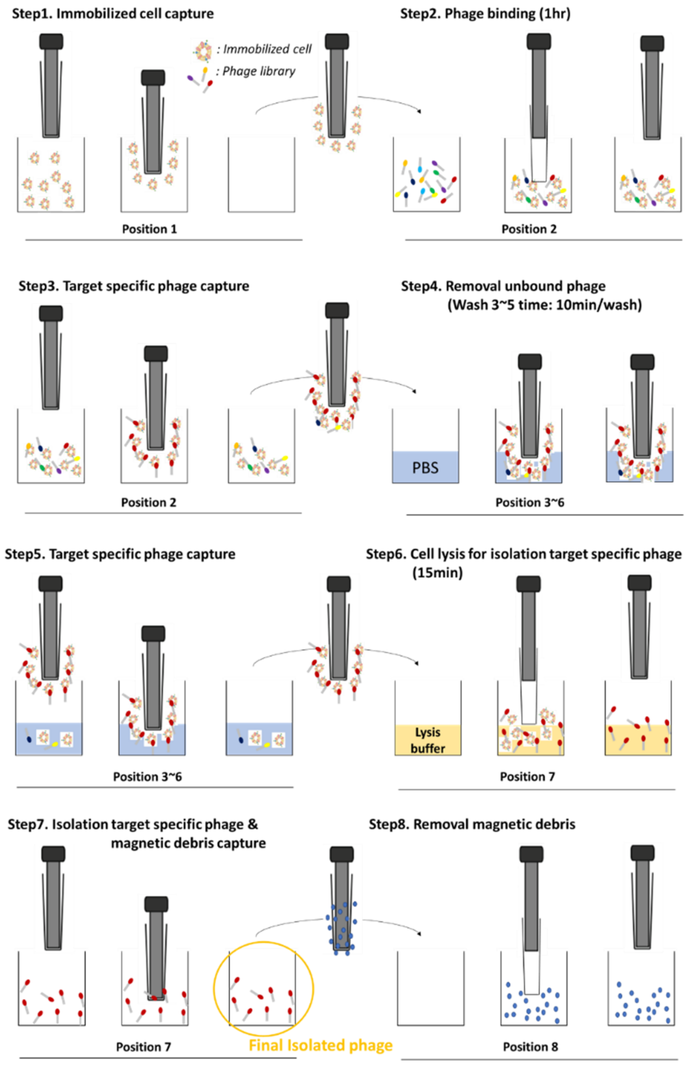

Supplementary figure 3. Fv modeling structure of 6 clones (VH-VL). The CDR-H3 loop was annotated with different colors. A1D06 (yellow), S2D05 (green), S3A06 (cyan), S3B09 (magenta), S1E12 (blues), and A1A10 (red).

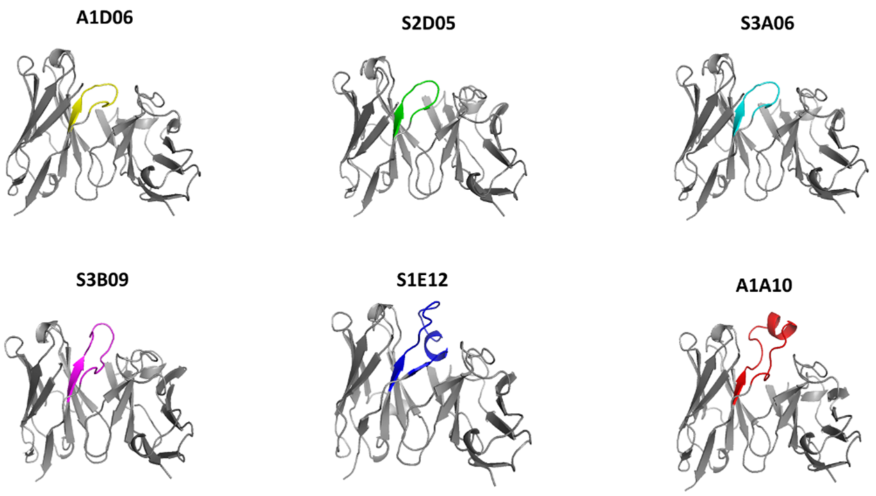

Supplementary figure 4. Schematic diagram of vector cloning for conversion of scFv to IgG1.

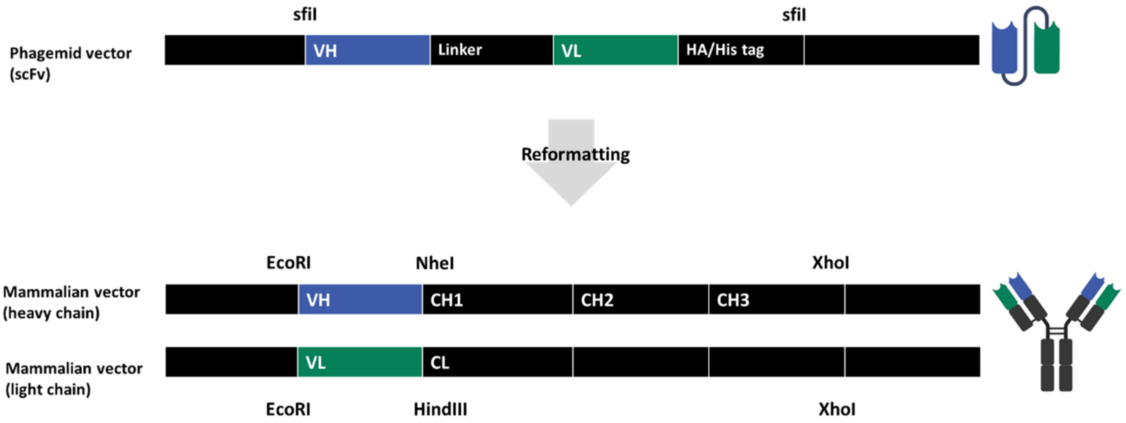

Supplementary figure 5. SPR kinetic analysis of clones to FGFR3 isotype and interspecies FGFR3 using Biacore 3000. SPR sensorgram mean Response Unit (RU; Resp. Diff.) values at five concentration conditions (100 nM, 50 nM, 25 nM, 12.5 nM, and 6.25 nM) for each antigen during association time 180 s and dissociation time 300 s.

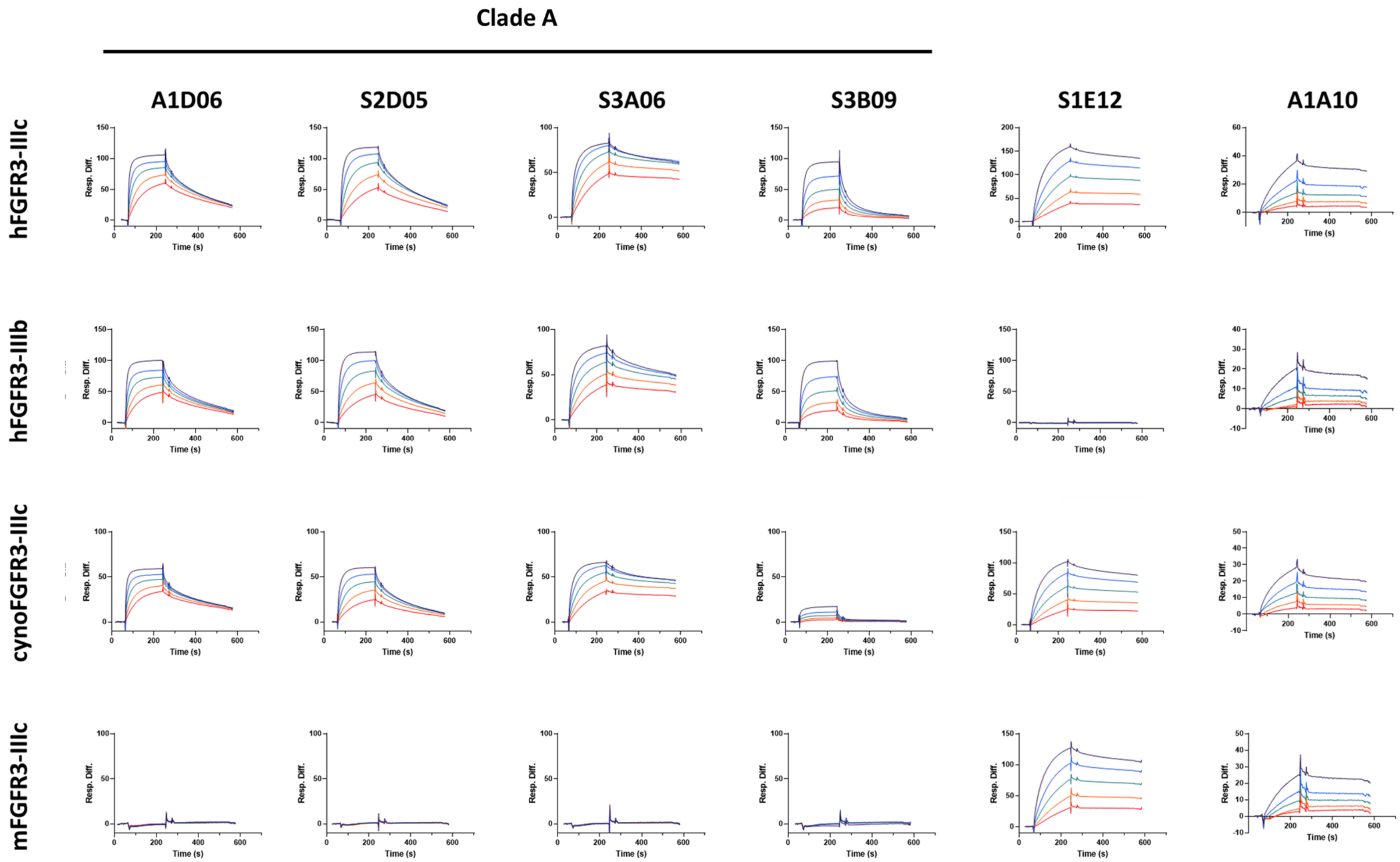

Supplement: Supplementary file 1 [file ijms-22-06240-s001.zip › ijms-1227926-supplementary.pdf]
